# Crystal structure of 1-phenyl-1*H*-pyrazolo­[3,4-*d*]pyrimidin-4(5*H*)-one

**DOI:** 10.1107/S205698902500934X

**Published:** 2025-10-28

**Authors:** Nilufar Akhmedova, Ilkhom Ortikov, Nilufar Khasanova, Kosim Zokhidov, Kambarali Turgunov, Burkhon Elmuradov

**Affiliations:** a S. Yunusov Institute of the Chemistry of Plant Substances Academy of Sciences of Uzbekistan Mirzo Ulugbek Str 77 Tashkent 100170 Uzbekistan; b Alfraganus University, Yukori Korakamysh str., 2, Tashkent 100190, Uzbekistan; c Samarkand State University, 15, University blv., 140104, Samarkand, Uzbekistan; d Turin Polytechnic University in Tashkent Kichik Khalka yuli str 17 100095 Tashkent Uzbekistan; Vienna University of Technology, Austria

**Keywords:** crystal structure, mol­ecular structure, 1-phenyl-1*H*-pyrazolo­[3,4-*d*]pyrimidin-4(5*H*)-one

## Abstract

Heating of 5-amino-1-phenyl-1*H*-pyrazole-4-carbo­nitrile in the presence of formic acid yielded the title compound, C_11_H_8_N_4_O. The pyrazolo­[3,4-*d*]pyrimidine ring system forms a dihedral angle of 34.72 (6)° with the phenyl ring. In the crystal, classical N—H⋯O and non-classical C—H⋯O and C—H⋯N inter­molecular inter­actions result in the formation of supra­molecular bands extending parallel to the *a* axis. Additional π⋯π inter­actions between pyrimidine and pyrazole rings, and C—H⋯π inter­actions between neighboring phenyl rings consolidate the packing.

## Abstract 2

The phenyl ring forms a dihedral angle of 34.72 (6)° with the mean plane of the pyrazolo­[3,4-*d*]pyrimidine ring system.

## 1. Chemical context

Cancer is one of the most difficult to treat and rapidly increasing diseases worldwide. The number of cancer types is increasing every year, which worries the World Health Organization and specialists in the field. One of the urgent tasks facing chemists and pharmacists is therefore to create and implement new, effective drugs against cancer. An analysis of the literature shows that pyrazolo­pyrimidines, a class of compounds we have selected for our research, are among the pharmaceutically active substances. Synthetic pyrazolo­pyrimidines are widely used in medicine. In particular, drugs based on these compounds are used to repair cells damaged by viruses, microbes, and cancer. Examples of such drugs include allopurinol, istrafilin, and ruxolitinib. Research in the field of pyrazolo­pyrimidines is advancing with the design, screening, synthesis, and biological evaluation of potential cancer drugs that inhibit tumor growth and induce apoptosis (He *et al.*, 2011 ▸; Gillespie *et al.*, 2008 ▸; Schenone *et al.*, 2004 ▸; Tintori *et al.*, 2015 ▸; Gaber *et al.*, 2022 ▸; Trivedi *et al.*, 2012 ▸). Among these, compounds containing a pyrazolo­[3,4-*d*]pyrimidine moiety exhibit broad anti­cancer activity *in vitro*. These derivatives are of further inter­est due to their high similarity to the adenine moiety of ATP. To obtain new pyrazolo­[3,4-*d*]pyrimidine-4-one derivatives, we carried out the reaction of 5-amino-1-phenyl-1*H*-pyrazole-4-carbo­nitrile with formic acid, yielding 1-phenyl-1*H*-pyrazolo­[3,4-*d*]pyrimidin-4(5*H*)-one, C_11_H_8_N_4_O, the crystal structure of which is reported here.
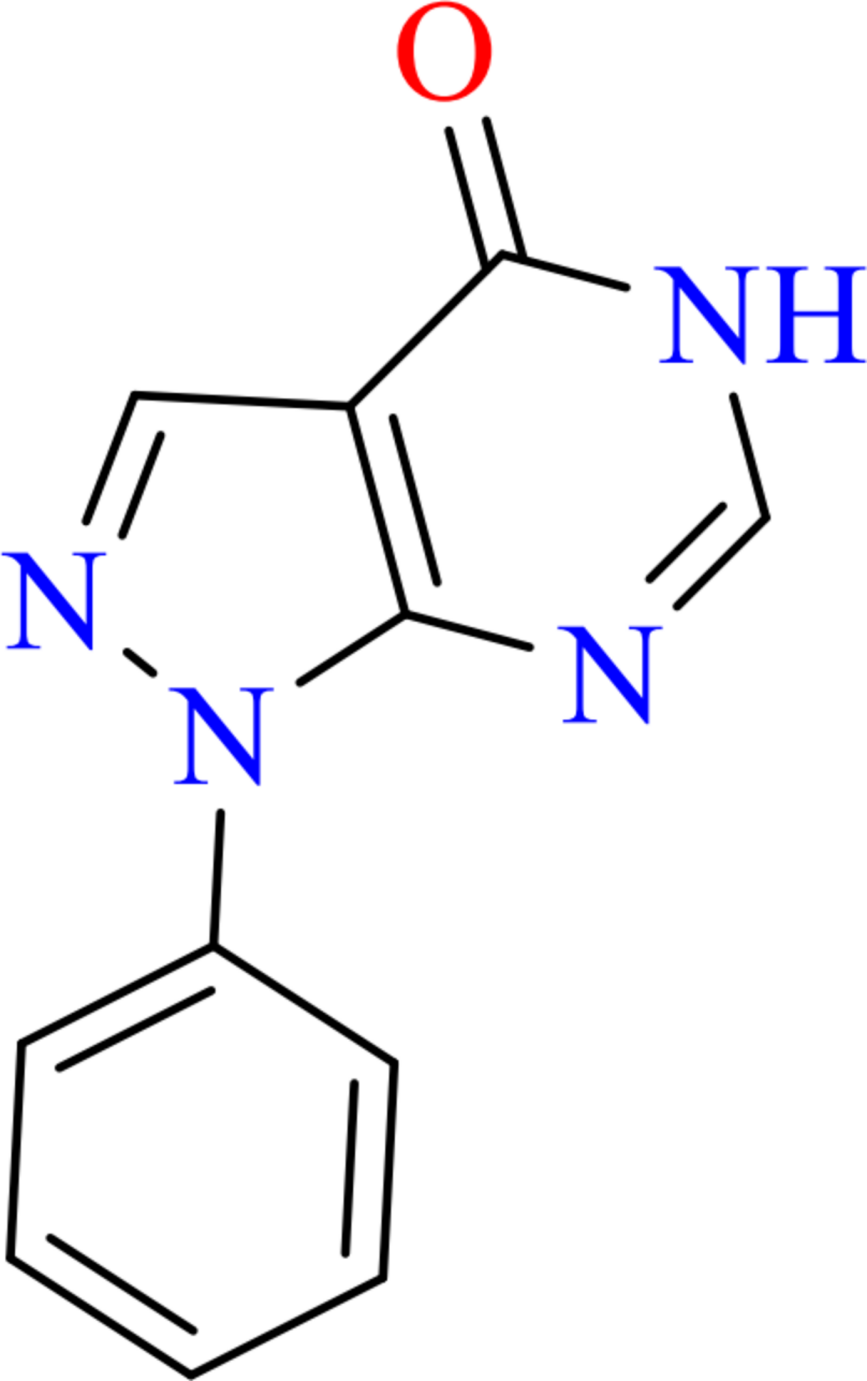


## 2. Structural commentary

**Figure 1:**
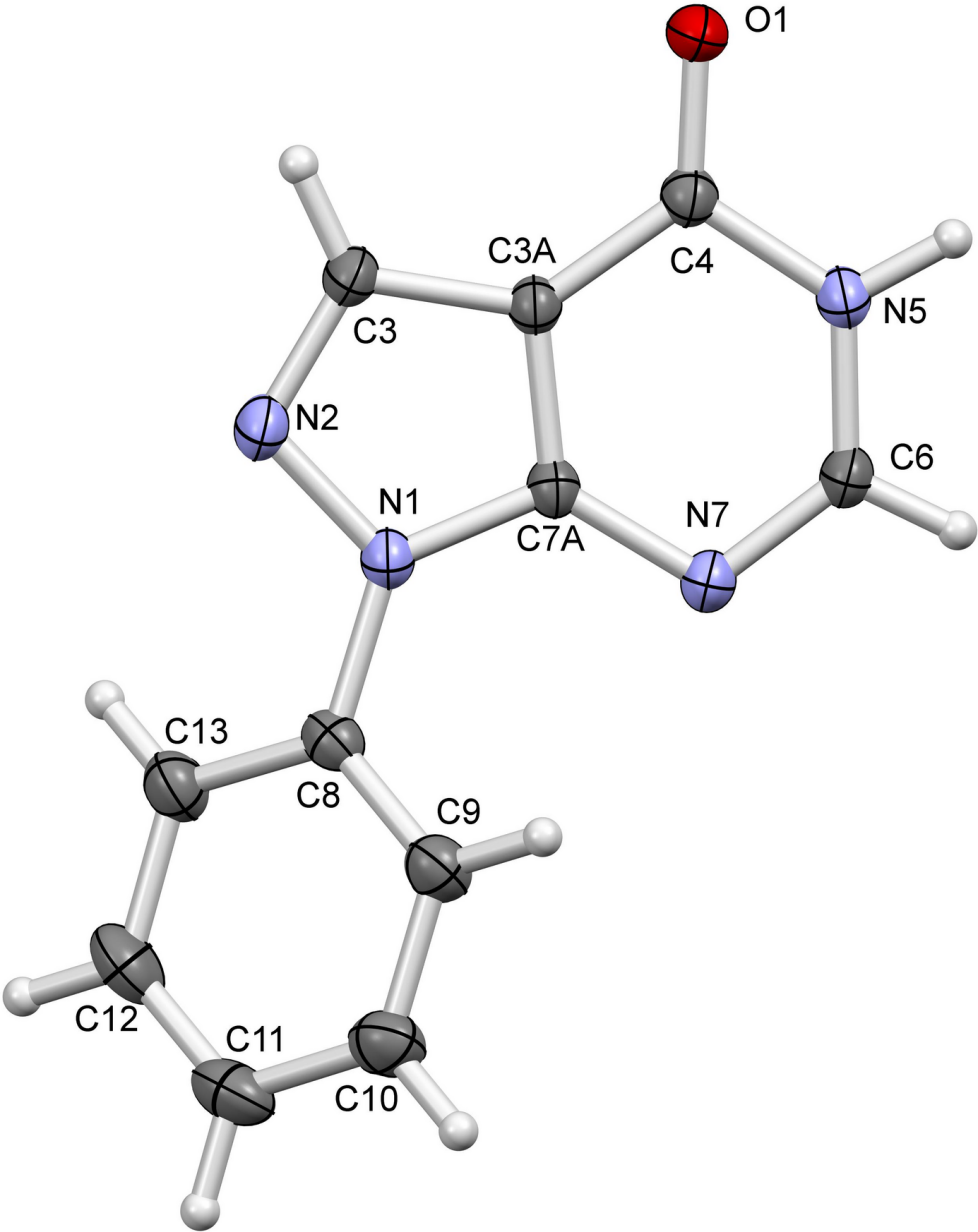
The mol­ecular structure of the title compound, with displacement ellipsoids drawn at the 50% probability level.

The asymmetric unit consist of one formula unit (Fig. 1 ▸). Selected N—N and N—C bond lengths given in Table 1 ▸ correspond to those observed in related structures [Cambridge Structure Database (CSD; Groom *et al.*, 2016 ▸) refcodes CICPAG, NIFGUF, UBAVUS and VICGOD (Yathirajan *et al.*, 2007*a* ▸,*b* ▸; Wang *et al.*, 2021 ▸; Ferroni *et al.*, 1990 ▸, respectively)]. In the unsubstituted base, 1*H*-pyrazolo­[3,4-*d*]pyrimidin-4(5*H*)-one, the N—N and N—C bond lengths within the pyrazole ring are slightly shorter (N1—N2 = 1.363 Å, N1—C7*A* = 1.343 Å and N2—C3 = 1.313 Å; ALOPUR01, Tang *et al.*, 2023 ▸). The pyrazolo­[3,4-*d*]pyrimidine ring system is planar with an r.m.s. deviation of 0.012 Å for the ring atoms. The phenyl ring (C8–C13) forms a dihedral angle of 34.72 (6)° with the mean plane of the pyrazolo­[3,4-*d*]pyrimidine ring system.

## 3. Supra­molecular features

**Figure 2:**
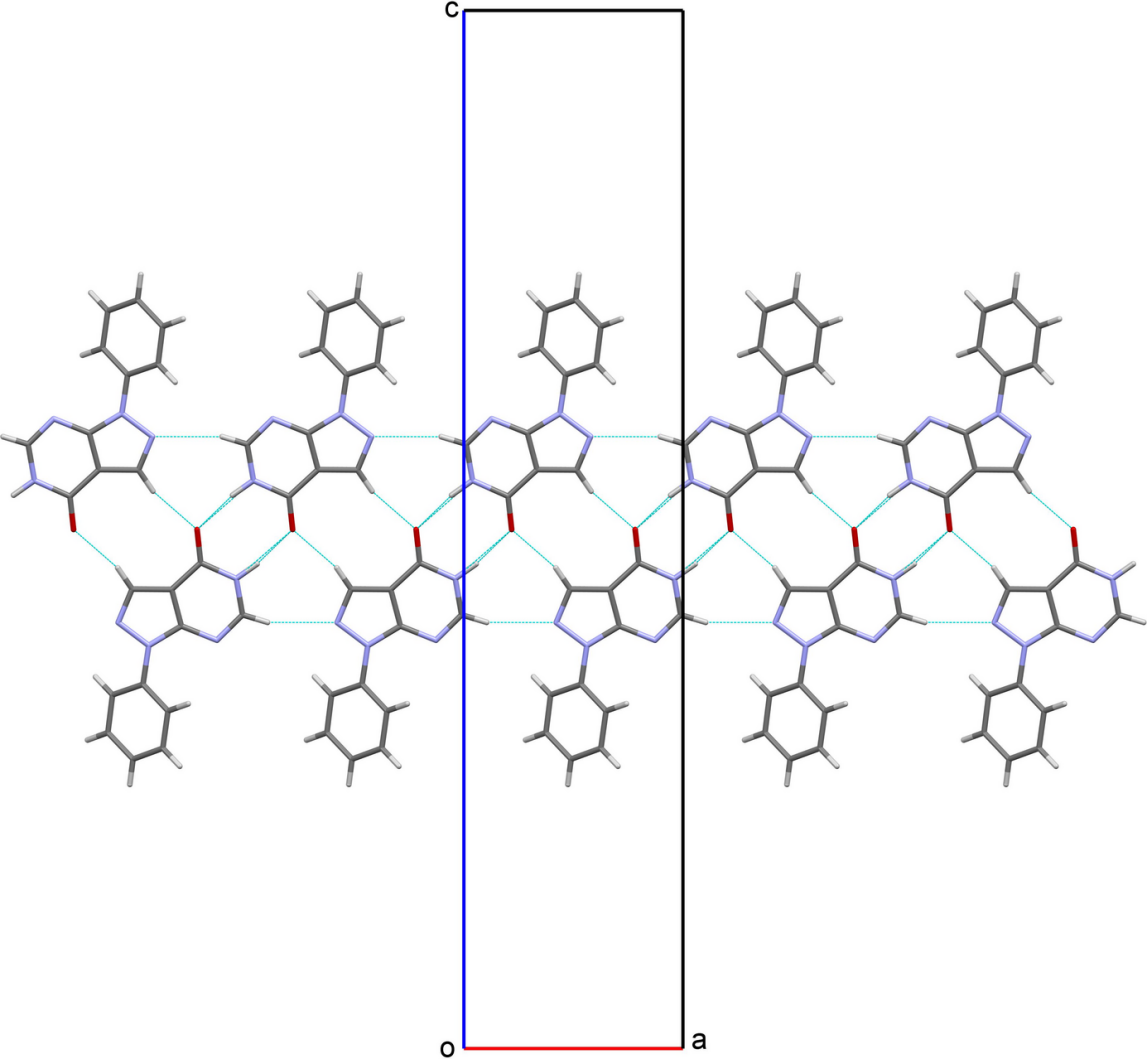
Formation of supra­molecular bands running parallel to the *a* axis.

**Figure 3:**
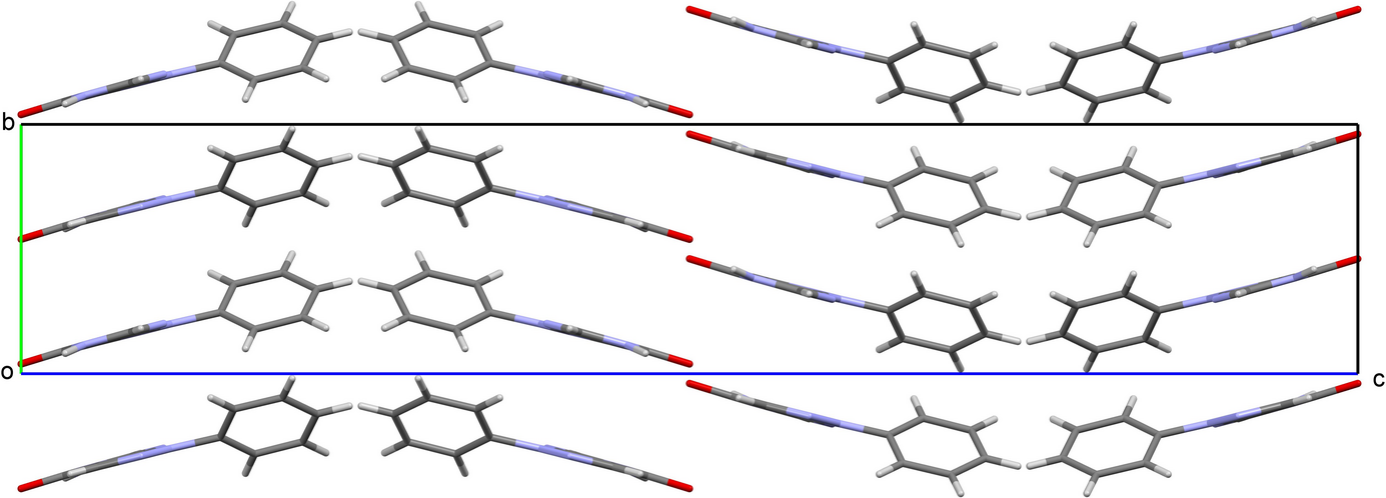
Packing of the supra­molecular bands along the *b* axis.

In the crystal of the title compound, inter­molecular N—H⋯O hydrogen bonds link the mol­ecules into centrosymmetric dimers, forming 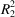
(8) motifs. Other centrosymmetric 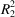
(10) motifs are formed by weak inter­molecular C—H⋯O bonds, and a weak C—H⋯N hydrogen bond participates in the formation of 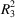
(8) ring motifs (Table 2 ▸). These hydrogen bonds generate supra­molecular bands running parallel to the *a* axis (Fig. 2 ▸). The bands are further packed along the *b-*axis direction through pairs of π–π stacking inter­actions (Fig. 3 ▸) [*Cg*2⋯*Cg*3^v^ and *Cg*3⋯*Cg*2^v^, centroid-to-centroid distances of 3.4644 (11) and 3.6444 (11) Å and a slippage of 0.860 and 1.471 Å, respectively; *Cg*2 is the pyrimidine ring centroid, *Cg*3 is the pyrazole ring centroid; symmetry code: (v) 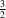 − *x*, −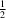 + *y*, *z*). In addition, an inter­molecular C_ar_—H⋯π inter­action is observed between the phenyl ring and the centroid of a neighboring phenyl ring (*Cg*1; Table 2 ▸).

## 4. Database survey

A search of the Cambridge Structural Database (CSD; updated 16 May 2025; Groom *et al.*, 2016 ▸) revealed 91 relevant entries containing the 1*H*-pyrazolo­[3,4-*d*]pyrimidin-4(5*H*)-one moiety. Two of these, namely ALOPUR (Prusiner & Sundaralingam, 1972 ▸) and ALOPUR01 (Tang *et al.*, 2023 ▸), correspond to allopurinol, *i.e*., the 1*H*-pyrazolo­[3,4-*d*]pyrimidin-4(5*H*)-one structure. Two other entries, SUTRUX and SUTSAE (Dai *et al.*, 2020 ▸), are co-crystals of allopurinol. The structures of allopurinol salts with maleic and oxalic acids were determined by powder X-ray diffraction analysis (Varsa *et al.*, 2023 ▸). Among the retrieved entries, more than 20 correspond to metal-containing structures.

## 5. Synthesis and crystallization

**Figure 4:**
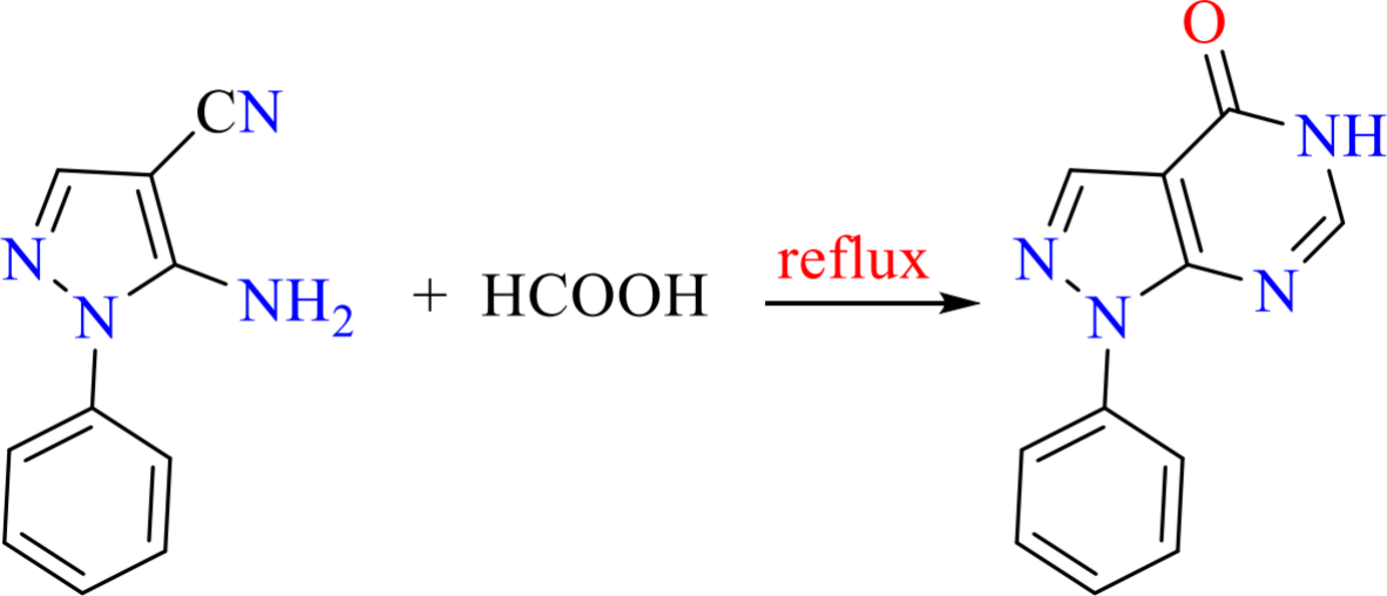
Synthesis scheme for the title compound.

**Figure 5:**
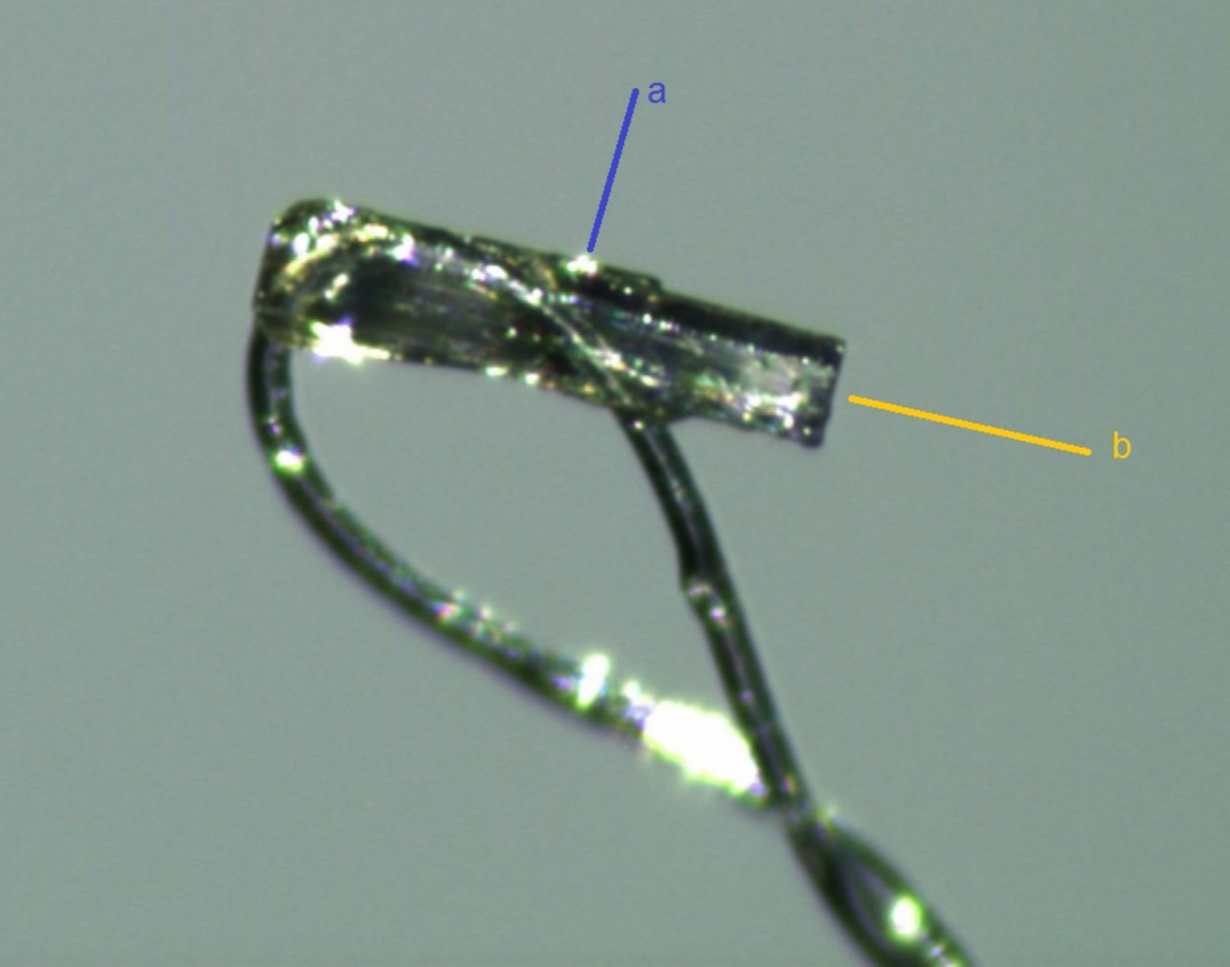
Screenshot from the face-indexing procedure showing the *a* and *b* axes of the rod-shaped crystal.

The reaction scheme is displayed in Fig. 4 ▸. Amino-1-phenyl-1*H*-pyrazole-4-carbo­nitrile (4. 2 g, 10.8 mmol) was heated in 10 ml of formic acid at 383–385 K for 6 h. Then, the mixture was poured into a 250 ml beaker of ice–water and the resulting precipitate was filtered off and dried. Recrystallization from ethanol yielded 1.98 g (86%) of 1-phenyl-1*H*-pyrazolo­[3,4-*d*]pyrimidin-4(5*H*)-one in form of rod-shaped crystals (Fig. 5 ▸), m.p. 577–578 K, *R*_f_ = 0.38.

## 6. Refinement

Crystal data, data collection and structure refinement details are summarized in Table 3 ▸. Hydrogen atoms bonded to carbon atoms were placed in calculated positions and refined to ride on their parent atoms with C—H = 0.93 Å and *U*_iso_(H) = 1.2*U*_eq_(C). The H atom attached to N5 was located in a difference-Fourier map, and its coordinates and isotropic displacement parameter were refined freely.

## Supplementary Material

Crystal structure: contains datablock(s) I. DOI: 10.1107/S205698902500934X/wm5773sup1.cif

Structure factors: contains datablock(s) I. DOI: 10.1107/S205698902500934X/wm5773Isup2.hkl

Supporting information file. DOI: 10.1107/S205698902500934X/wm5773Isup3.cml

CCDC reference: 2497282

Additional supporting information:  crystallographic information; 3D view; checkCIF report

## Figures and Tables

**Table 1:** Selected bond lengths (Å)

N1—C7*A*	1.358 (2)	N2—C3	1.321 (2)
N1—N2	1.376 (2)	N5—C6	1.355 (2)
N1—C8	1.428 (2)	N7—C6	1.304 (2)

**Table 2:** Hydrogen-bond geometry (Å, °) *Cg*1 is the centroid of the C8–C13 ring.

*D*—H⋯*A*	*D*—H	H⋯*A*	*D*⋯*A*	*D*—H⋯*A*
N5—H5⋯O1^i^	0.94 (3)	1.94 (3)	2.862 (2)	166 (2)
C3—H3⋯O1^ii^	0.93	2.28	3.180 (2)	162
C6—H6⋯N2^iii^	0.93	2.48	3.374 (2)	162
C9—H9⋯*Cg*1^iv^	0.93	2.94	3.767 (2)	150

Symmetry codes: (i) 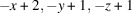; (ii) 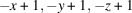; (iii) 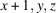; (iv) 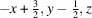.

**Table 3:** Experimental details

Crystal data	
Chemical formula	C_11_H_8_N_4_O
*M*_r_	212.21
Crystal system, space group	Orthorhombic, *Pbca*
Temperature (K)	293
*a*, *b*, *c* (Å)	7.7822 (2), 6.8848 (2), 36.8514 (10)
*V* (Å^3^)	1974.46 (9)
*Z*	8
Radiation type	Cu *K*α
μ (mm^−1^)	0.81
Crystal size (mm)	0.44 × 0.12 × 0.05

Data collection	
Diffractometer	Bruker D8 VENTURE dual wavelength Mo/Cu
Absorption correction	Numerical (*SADABS*; Krause *et al.*, 2015 ▸)
*T*_min_, *T*_max_	0.659, 0.753
No. of measured, independent and observed [*I* > 2σ(*I*)] reflections	20676, 1807, 1619
*R*_int_	0.054
(sin θ/λ)_max_ (Å^−1^)	0.602

Refinement	
*R*[*F*^2^ > 2σ(*F*^2^)], *wR*(*F*^2^), *S*	0.046, 0.120, 1.13
No. of reflections	1807
No. of parameters	150
H-atom treatment	H atoms treated by a mixture of independent and constrained refinement
Δρ_max_, Δρ_min_ (e Å^−3^)	0.25, −0.19

Computer programs: *APEX5* and *SAINT* (Bruker, 2023 ▸), *SHELXT* (Sheldrick, 2015*a* ▸), *SHELXL* (Sheldrick, 2015*b* ▸), *Mercury* (Macrae *et al.*, 2020 ▸) and *publCIF* (Westrip, 2010 ▸).

## supplementary crystallographic information

### 1-Phenyl-1H-pyrazolo[3,4-d]pyrimidin-4(5H)-one Crystal data

**Table:**

C_11_H_8_N_4_O	*D*_x_ = 1.428 Mg m^−^^3^
*M**_r_* = 212.21	Melting point: 577(2) K
Orthorhombic, *P**b**c**a*	Cu *K*α radiation, λ = 1.54178 Å
*a* = 7.7822 (2) Å	Cell parameters from 3849 reflections
*b* = 6.8848 (2) Å	θ = 7.2–66.9°
*c* = 36.8514 (10) Å	µ = 0.81 mm^−^^1^
*V* = 1974.46 (9) Å^3^	*T* = 293 K
*Z* = 8	Prism, colourless
*F*(000) = 880	0.44 × 0.12 × 0.05 mm

### 1-Phenyl-1H-pyrazolo[3,4-d]pyrimidin-4(5H)-one Data collection

**Table:**

Bruker D8 VENTURE dual wavelength Mo/Cu diffractometer	1807 independent reflections
Radiation source: microfocus X-ray source, Incoatec IµS 3.0 Microfocus Source	1619 reflections with *I* > 2σ(*I*)
Mirror monochromator	*R*_int_ = 0.054
ω–φ scans	θ_max_ = 68.1°, θ_min_ = 7.2°
Absorption correction: numerical (*SADABS*; Krause *et al.*, 2015)	*h* = −9→9
*T*_min_ = 0.659, *T*_max_ = 0.753	*k* = −8→8
20676 measured reflections	*l* = −44→44

### 1-Phenyl-1H-pyrazolo[3,4-d]pyrimidin-4(5H)-one Refinement

**Table:**

Refinement on *F*^2^	Hydrogen site location: mixed
Least-squares matrix: full	H atoms treated by a mixture of independent and constrained refinement
*R*[*F*^2^ > 2σ(*F*^2^)] = 0.046	*w* = 1/[σ^2^(*F*_o_^2^) + (0.0495*P*)^2^ + 0.8037*P*] where *P* = (*F*_o_^2^ + 2*F*_c_^2^)/3
*wR*(*F*^2^) = 0.120	(Δ/σ)_max_ < 0.001
*S* = 1.13	Δρ_max_ = 0.25 e Å^−^^3^
1807 reflections	Δρ_min_ = −0.19 e Å^−^^3^
150 parameters	Extinction correction: SHELXL-2014/7 (Sheldrick 2015b), Fc^*^=kFc[1+0.001xFc^2^λ^3^/sin(2θ)]^-1/4^
0 restraints	Extinction coefficient: 0.0042 (6)

### 1-Phenyl-1H-pyrazolo[3,4-d]pyrimidin-4(5H)-one Special details

**Table:**

Geometry. All esds (except the esd in the dihedral angle between two l.s. planes) are estimated using the full covariance matrix. The cell esds are taken into account individually in the estimation of esds in distances, angles and torsion angles; correlations between esds in cell parameters are only used when they are defined by crystal symmetry. An approximate (isotropic) treatment of cell esds is used for estimating esds involving l.s. planes.

### 1-Phenyl-1H-pyrazolo[3,4-d]pyrimidin-4(5H)-one Fractional atomic coordinates and isotropic or equivalent isotropic displacement parameters (Å^2^)

**Table:**

	*x*	*y*	*z*	*U*_iso_*/*U*_eq_	
O1	0.78192 (17)	0.5391 (2)	0.49996 (3)	0.0534 (4)	
N1	0.56134 (18)	0.6999 (2)	0.38824 (4)	0.0416 (4)	
N2	0.42164 (19)	0.6696 (3)	0.41042 (5)	0.0489 (4)	
N5	0.9606 (2)	0.6158 (2)	0.45274 (4)	0.0450 (4)	
H5	1.056 (3)	0.582 (3)	0.4669 (6)	0.064 (7)*	
N7	0.87219 (19)	0.7013 (2)	0.39334 (4)	0.0434 (4)	
C3	0.4855 (2)	0.6268 (3)	0.44265 (5)	0.0446 (5)	
H3	0.4195	0.5980	0.4630	0.053*	
C3A	0.6660 (2)	0.6305 (3)	0.44230 (5)	0.0392 (4)	
C4	0.7984 (2)	0.5914 (3)	0.46802 (5)	0.0415 (4)	
C6	0.9895 (2)	0.6677 (3)	0.41782 (5)	0.0431 (5)	
H6	1.1035	0.6805	0.4106	0.052*	
C7A	0.7101 (2)	0.6778 (2)	0.40708 (5)	0.0371 (4)	
C8	0.5349 (2)	0.7457 (3)	0.35086 (5)	0.0442 (5)	
C9	0.6491 (3)	0.6783 (3)	0.32524 (6)	0.0538 (5)	
H9	0.7413	0.6008	0.3321	0.065*	
C10	0.6254 (3)	0.7269 (4)	0.28933 (6)	0.0640 (6)	
H10	0.7030	0.6827	0.2720	0.077*	
C11	0.4895 (4)	0.8388 (4)	0.27881 (7)	0.0696 (7)	
H11	0.4747	0.8705	0.2545	0.083*	
C12	0.3746 (4)	0.9045 (4)	0.30437 (7)	0.0718 (7)	
H12	0.2817	0.9803	0.2972	0.086*	
C13	0.3959 (3)	0.8587 (3)	0.34074 (6)	0.0585 (6)	
H13	0.3182	0.9031	0.3580	0.070*	

### 1-Phenyl-1H-pyrazolo[3,4-d]pyrimidin-4(5H)-one Atomic displacement parameters (Å^2^)

**Table:**

	*U*^11^	*U*^22^	*U*^33^	*U*^12^	*U*^13^	*U*^23^
O1	0.0433 (8)	0.0746 (10)	0.0423 (7)	−0.0024 (7)	−0.0020 (6)	0.0144 (6)
N1	0.0323 (8)	0.0477 (9)	0.0448 (9)	0.0009 (6)	−0.0018 (6)	0.0030 (6)
N2	0.0320 (8)	0.0594 (10)	0.0554 (10)	−0.0011 (7)	0.0015 (7)	0.0061 (8)
N5	0.0341 (8)	0.0538 (9)	0.0471 (9)	−0.0006 (7)	−0.0045 (7)	0.0068 (7)
N7	0.0336 (8)	0.0521 (9)	0.0446 (9)	0.0012 (7)	0.0013 (6)	0.0027 (7)
C3	0.0334 (9)	0.0531 (11)	0.0472 (10)	−0.0011 (8)	0.0032 (8)	0.0065 (8)
C3A	0.0331 (9)	0.0417 (9)	0.0427 (10)	0.0002 (7)	−0.0015 (7)	0.0029 (7)
C4	0.0369 (9)	0.0427 (10)	0.0449 (10)	−0.0007 (7)	−0.0004 (7)	0.0028 (7)
C6	0.0314 (9)	0.0516 (10)	0.0464 (10)	−0.0002 (8)	0.0016 (8)	0.0028 (8)
C7A	0.0318 (9)	0.0366 (9)	0.0428 (9)	−0.0004 (7)	−0.0023 (7)	0.0006 (7)
C8	0.0473 (10)	0.0412 (9)	0.0441 (10)	−0.0039 (8)	−0.0090 (8)	0.0035 (8)
C9	0.0563 (12)	0.0558 (12)	0.0491 (12)	0.0041 (10)	−0.0073 (9)	−0.0021 (9)
C10	0.0704 (15)	0.0753 (15)	0.0464 (12)	−0.0061 (12)	−0.0033 (11)	−0.0041 (10)
C11	0.0880 (18)	0.0724 (15)	0.0483 (12)	−0.0084 (13)	−0.0192 (12)	0.0098 (11)
C12	0.0732 (16)	0.0726 (15)	0.0696 (16)	0.0122 (13)	−0.0266 (13)	0.0112 (12)
C13	0.0536 (12)	0.0649 (13)	0.0571 (13)	0.0087 (11)	−0.0106 (10)	0.0011 (10)

### 1-Phenyl-1H-pyrazolo[3,4-d]pyrimidin-4(5H)-one Geometric parameters (Å, º)

**Table:**

O1—C4	1.237 (2)	C3A—C4	1.426 (2)
N1—C7A	1.358 (2)	C6—H6	0.9300
N1—N2	1.376 (2)	C8—C9	1.377 (3)
N1—C8	1.428 (2)	C8—C13	1.384 (3)
N2—C3	1.321 (2)	C9—C10	1.377 (3)
N5—C6	1.355 (2)	C9—H9	0.9300
N5—C4	1.392 (2)	C10—C11	1.365 (4)
N5—H5	0.94 (3)	C10—H10	0.9300
N7—C6	1.304 (2)	C11—C12	1.376 (4)
N7—C7A	1.369 (2)	C11—H11	0.9300
C3—C3A	1.405 (2)	C12—C13	1.387 (3)
C3—H3	0.9300	C12—H12	0.9300
C3A—C7A	1.381 (2)	C13—H13	0.9300
			
C7A—N1—N2	110.66 (14)	N1—C7A—C3A	107.17 (15)
C7A—N1—C8	129.83 (16)	N7—C7A—C3A	127.16 (16)
N2—N1—C8	119.51 (15)	C9—C8—C13	120.60 (19)
C3—N2—N1	105.69 (14)	C9—C8—N1	119.61 (17)
C6—N5—C4	124.55 (16)	C13—C8—N1	119.79 (18)
C6—N5—H5	117.5 (15)	C8—C9—C10	119.4 (2)
C4—N5—H5	117.6 (15)	C8—C9—H9	120.3
C6—N7—C7A	111.62 (15)	C10—C9—H9	120.3
N2—C3—C3A	111.35 (17)	C11—C10—C9	120.9 (2)
N2—C3—H3	124.3	C11—C10—H10	119.5
C3A—C3—H3	124.3	C9—C10—H10	119.5
C7A—C3A—C3	105.13 (16)	C10—C11—C12	119.7 (2)
C7A—C3A—C4	119.32 (16)	C10—C11—H11	120.2
C3—C3A—C4	135.49 (18)	C12—C11—H11	120.2
O1—C4—N5	120.94 (16)	C11—C12—C13	120.6 (2)
O1—C4—C3A	127.74 (17)	C11—C12—H12	119.7
N5—C4—C3A	111.31 (16)	C13—C12—H12	119.7
N7—C6—N5	125.98 (17)	C8—C13—C12	118.8 (2)
N7—C6—H6	117.0	C8—C13—H13	120.6
N5—C6—H6	117.0	C12—C13—H13	120.6
N1—C7A—N7	125.67 (16)		
			
C7A—N1—N2—C3	−0.7 (2)	C6—N7—C7A—C3A	1.9 (3)
C8—N1—N2—C3	178.78 (16)	C3—C3A—C7A—N1	0.0 (2)
N1—N2—C3—C3A	0.7 (2)	C4—C3A—C7A—N1	177.64 (16)
N2—C3—C3A—C7A	−0.4 (2)	C3—C3A—C7A—N7	179.11 (18)
N2—C3—C3A—C4	−177.5 (2)	C4—C3A—C7A—N7	−3.2 (3)
C6—N5—C4—O1	178.24 (18)	C7A—N1—C8—C9	34.0 (3)
C6—N5—C4—C3A	−0.8 (3)	N2—N1—C8—C9	−145.37 (19)
C7A—C3A—C4—O1	−176.59 (18)	C7A—N1—C8—C13	−145.4 (2)
C3—C3A—C4—O1	0.2 (4)	N2—N1—C8—C13	35.2 (3)
C7A—C3A—C4—N5	2.3 (2)	C13—C8—C9—C10	1.0 (3)
C3—C3A—C4—N5	179.1 (2)	N1—C8—C9—C10	−178.44 (19)
C7A—N7—C6—N5	0.0 (3)	C8—C9—C10—C11	−0.7 (4)
C4—N5—C6—N7	−0.4 (3)	C9—C10—C11—C12	0.1 (4)
N2—N1—C7A—N7	−178.70 (17)	C10—C11—C12—C13	0.3 (4)
C8—N1—C7A—N7	1.9 (3)	C9—C8—C13—C12	−0.6 (3)
N2—N1—C7A—C3A	0.5 (2)	N1—C8—C13—C12	178.8 (2)
C8—N1—C7A—C3A	−178.97 (17)	C11—C12—C13—C8	0.0 (4)
C6—N7—C7A—N1	−179.11 (17)		

### 1-Phenyl-1H-pyrazolo[3,4-d]pyrimidin-4(5H)-one Hydrogen-bond geometry (Å, º)

*Cg*1 is the centroid of the C8–C13 ring.

**Table:**

*D*—H···*A*	*D*—H	H···*A*	*D*···*A*	*D*—H···*A*
N5—H5···O1^i^	0.94 (3)	1.94 (3)	2.862 (2)	166 (2)
C3—H3···O1^ii^	0.93	2.28	3.180 (2)	162
C6—H6···N2^iii^	0.93	2.48	3.374 (2)	162
C9—H9···*Cg*1^iv^	0.93	2.94	3.767 (2)	150

Symmetry codes: (i) −*x*+2, −*y*+1, −*z*+1; (ii) −*x*+1, −*y*+1, −*z*+1; (iii) *x*+1, *y*, *z*; (iv) −*x*+3/2, *y*−1/2, *z*.
